# Integrating Green Care Initiatives into Conventional Health Systems: Which Governance Dimensions Can Guide This Process?

**DOI:** 10.3390/ijerph22020202

**Published:** 2025-01-30

**Authors:** Alessandra Rigo, Elena Pisani, Laura Secco

**Affiliations:** Department of Land, Environment, Agriculture and Forestry (TESAF), University of Padova, Agripolis—Viale dell’Università 16, 35020 Legnaro, Italy

**Keywords:** governance, green care initiatives, healthcare, literature review, nature-based health initiatives, green and blue areas

## Abstract

Green Care initiatives (GCIs) encompass various interventions that support physical, mental, and social well-being through interaction with nature. Integrating GCIs into conventional healthcare systems is a complex task that requires multi-actor and multi-level governance efforts. This study examines, through a systematized literature review, the relevant governance dimensions to facilitate the inclusion of GCIs in traditional care pathways. From the analysis of the 36 selected studies, four key dimensions were identified: organizational structure, knowledge, legitimacy, and decentralization. The analysis highlights the need to strengthen the responsibility of Green Care actors as healthcare service providers, enhance local authorities’ role in creating new integrated service delivery networks, combine different knowledge perspectives, and legitimize non-healthcare actors. Recommendations are made to address these governance aspects to facilitate the integration of GCIs and channel their benefits in prevention and health promotion. Adopting an adequate governance framework is fundamental for mainstreaming GCIs in current healthcare systems.

## 1. Introduction

Engagement in nature-based activities such as gardening, walking in a park, or exploring a forest by activating the use of the senses has been demonstrated to enhance both physical and mental well-being [1,2,3]. There is a wide range of terms to denote nature-based health initiatives [4,5], including—among others—social farming, forest bathing, therapeutic horticulture, animal-assisted interventions, and ecotherapy [6,7,8]. For the purposes of this study, we chose the term “Green Care initiatives” (GCIs) as an umbrella term denoting structured interventions promoting physical, mental, and social health through consciously interacting with nature [9,10,11,12].

Notwithstanding the increasing empirical evidence supporting the health benefits of being in nature, the majority of national health institutions have yet to fully acknowledge the potential of GCIs in the context of health and social service provision [13,14,15]. This lack of recognition may be attributed to the current focus of Green Care (GC) evidence on biomedical effects, coupled with a limited understanding of the institutional and actor landscapes, as well as the governance arrangements necessary for the mainstreaming of GCIs into health systems. Notable exceptions include Japan, South Korea, and Canada [16,17,18,19], where GC has been fully integrated into national health systems, with “green prescriptions” being implemented by numerous medical practitioners and patients.

GCIs can be conceptualized as the outcome of multi-level interactions between public authorities and actors from diverse sectors, including health, social services, education, and green/blue area management [12,20,21]. These actors are crucial as their actions and management interventions can facilitate the provision of accessible, safe, and appropriate green or blue spaces, including urban parks, forests, lakes, coasts, and protected areas, where individuals can engage in positive and effective nature-based health experiences. Concurrently, institutional support from policymakers is imperative to legitimize the integration of GCIs into conventional health systems [22,23,24].

While the adoption of innovative approaches to health service delivery necessitates a more profound consideration of governance in the health sector [22,25], the integration of non-health and nature-related actors introduces additional complexity. In conjunction with emerging caregiver and social service profiles (e.g., certified forest bathing guides, urban horticulture-based social inclusion organizations), including nature in healthcare services entails engagement with green/blue areas and natural space managers [26].

The successful implementation of GCIs requires not only multi-level and multi-sector interactions, but also shared value systems and novel governance arrangements, thereby establishing a new domain for social innovation [27]. To effectively navigate and manage this complexity, which embraces the novelty of collaboration and mutual understanding between actors in human health and natural settings, it is essential to identify, analyze, describe, classify, and understand its various dimensions from a governance perspective, building on existing knowledge (scientific publications).

By adopting an analytical approach and through a systematized literature review, this study aims to identify the salient governance-related aspects pertinent to GCIs and to highlight the necessary changes in these aspects for the integration of GCIs into conventional health systems. The findings are intended to guide the actions of the health, nature management, and intermediary sectors. Our research is guided by the following questions: (i) What are the key governance dimensions to be considered? (ii) Who are the relevant actors involved? (iii) What governance changes are required for the mainstreaming of GCIs into conventional health systems?

The ultimate goal of this research is to delineate a potential pathway for integrating GCIs into conventional health systems and to lay the foundation for developing a set of indicators. These indicators will serve as valuable tools to guide relevant stakeholders and decision-makers in the design, implementation, and evaluation of health systems that effectively incorporate nature in actions to address escalating human health challenges.

The paper is organized as follows: the subsequent sections elucidate guiding concepts and ideas, followed by a description of materials and methods. Then, we present the results and discussion by focusing on recommendations, followed by concluding remarks.

### 1.1. Growing Challenges for Conventional Health Systems

While life expectancy has increased significantly in recent decades [28,29], this trend has not been accompanied by a commensurate increase in healthy life years [30]. The confluence of population aging, consuming habits, and deteriorating environmental conditions, including the progressive loss of green spaces, directly impacts the global burden of Non-Communicable Diseases (NCDs) [23,31,32,33].

There is extensive literature documenting associations between NCDs and mental disorders [34,35,36]. Concurrently, various societal and environmental challenges, including the COVID-19 pandemic, war conflicts, the climate crisis, unemployment, and escalating living costs, have exacerbated mental health issues [37,38,39]. Furthermore, unhealthy lifestyles and higher use of digital devices increase loneliness, anxiety, and depression, especially among younger age groups [40,41].

In addition to the health impacts on individuals, mental health problems have economic and social consequences. The economic burden of mental health disorders is estimated to exceed 4% of the European Gross Domestic Product (GDP), equating to approximately EUR 600 billion [42]. Despite this significant economic impact, approximately half of the EU’s youth population (49%) report unmet mental healthcare needs, a proportion markedly higher than that observed in older adults (23%) [43]. The World Health Organization (WHO) [44] highlights an additional concerning trend: individuals with severe mental health conditions experience premature mortality due to preventable mental health conditions that could be addressed through relatively low cost interventions. The spread of chronic diseases, coupled with population aging, is generating novel health needs that exacerbate the financial strain on public health systems and the broader economy, potentially compromising equitable and affordable access to care [28,45]. The gap between people needing care and those with access to care services is critical, underscoring the urgent need for implementing effective prevention strategies to strengthen healthcare provision for vulnerable groups and the general population [46,47,48].

### 1.2. Embracing the Complexity of Health System Governance

Governance plays a pivotal role in ensuring “the careful and responsible management of the well-being of the population” [49] (p. 45) and is crucial in strengthening healthcare systems [50,51]. The concept of governance in the health sector has evolved significantly, encompassing various interpretations such as leadership, regulation, oversight, and stewardship [50,51,52]. Following the World Bank’s call for ’better governance’ (1999), health governance has been increasingly framed as the steering and regulatory function of national administrators within health systems, aimed at improving performance and health outcomes [49,52,53,54].

Scholars have proposed different approaches to facilitate the understanding of governance. In Europe, Kjær (2004) advocated for an institutional approach, i.e., focused on the setting, application, and enforcement of ‘rules’, according to which the institutional context “provides a common ground to all of the different perceptions of governance” [55] (p. 10). This perspective aligns with the World Health Report 2000 [49], which delineates organizations (e.g., individual providers, hospitals, clinics) as the players, interventions as the objects (e.g., care services or initiatives), and institutions as the formal and informal rules governing interactions. More recently, researchers like Baez Camargo and Jacobs (2011) [56] and Savigny and Adams (2009) [57] have promoted a systems thinking approach, emphasizing the synergies and interactions among all health system actors and highlighting governance’s cross-cutting nature.

While these conceptualizations provide valuable insights into key aspects of health sector governance (e.g., power distribution, leadership, administrative rules, competences, structural hierarchy, and networks), the methods for assessing governance, and how it contributes in terms of health outcomes, remain largely unexplored [58,59,60,61]. In response to this challenge, leading international organizations such as the Organization for Economic Cooperation and Development (OECD), World Bank, and US Agency for International Development (USAID) have contributed to progress in this direction. This is reflected in efforts to operationalize the term governance into dimensions and components and measurable indicators aimed at assessing governance in health systems [62,63] through either rule-based or outcome-based indicators [51,60].

Based on these premises, effectively integrating GCIs into traditional health systems requires a thorough understanding of key governance aspects, specified into dimensions and components and assessed using specific indicators. This approach is essential for evaluating the contribution of GCIs in terms of health outcomes.

### 1.3. Integrating GCIs into Conventional Health Systems: A Possible Conceptual Framework on Governance

Analyzing health system governance becomes particularly complex when considering the variety of stakeholders and sectors engaged in GCIs, each with their own perception of key rules or outcomes. These actors may include private, public, or community-based landowners, urban planners, rural development agencies, nature conservation activists, park managers, consultants, and others. Their perspectives may range from focusing on landscapes to small-scale land units, with different conceptions of nature and its value, spanning from the dominant instrumental approach to the emerging relational one [64,65]. The diversity is not limited to actors, but extends to environments and natural settings, which vary significantly across countries. This heterogeneity accentuates the need to identify common governance dimensions to facilitate the integration of nature and related GCIs into conventional healthcare systems on a broader scale.

**Figure 1:**
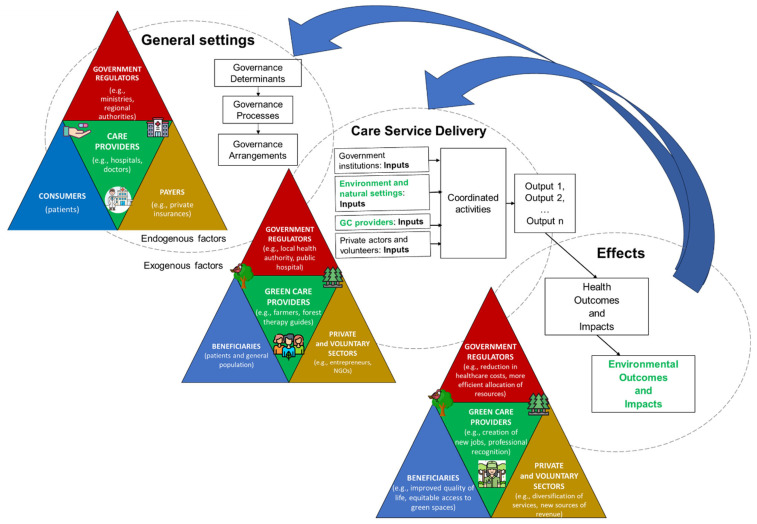
Representation of our conceptual framework for the integration of GCIs into the health governance system (Source: own elaboration based on [60,66]).

For systematically analyzing the complex interactions between GCIs, health systems, and governance, we propose a conceptual framework on the health governance system that includes GCIs as an integral part of care service delivery (Figure 1). Our approach is informed by the theoretical frameworks proposed by [56,60,61,66]. We designed our framework adopting a Theory of Change (ToC) approach based on the cause–effect vision, i.e., assuming that certain decisions, inputs, and activities will lead to expected outputs and, consequently, to positive results. This logic helps explore how GCIs can convert inputs, activities, and outputs into benefits in terms of health and environmental outcomes and impacts. It is important to note that, at this stage, we do not assess the effectiveness of GCIs in achieving outcomes. Instead, we lay the groundwork for a normative approach to be developed in the future, which will evaluate the performance of nature-based healthcare systems, considering both human health and positive outcomes for nature management.

Mapping the “General settings” enables the identification of conditions that delineate the normal functioning of the health governance system and, consequently, outlines aspects to consider when integrating GCIs into conventional health systems. Within the health governance system, Savedoff and Hussmann (2006) [66] categorized the numerous actors into five groups: (i) government regulators, (ii) payers, (iii) providers, (iv) consumers, and (v) suppliers. We have adapted this classification to visualize key actors typically involved in the health governance system, illustrate how GC providers interact with these actors, and anticipate the effects on each category following GCI implementation.

The multi-level and multi-sector interactions among actors within the governance system are shaped by both endogenous factors, such as structural and relational contingencies (e.g., trust, goals consensus), and exogenous factors, including form, inception type (voluntary or mandated), and the developmental stage of involved actor networks [67]. Moreover, governance determinants, processes, and arrangements influence the general settings for governance. Governance determinants, in particular, influence actors’ individual or institutional behavior in fulfilling their roles and responsibilities [60,68] by providing incentives and constraints [49,56].

Governance determinants, processes, and arrangements also influence the “Care Service Delivery” chain, where GCI integration actually occurs. This chain comprises the inputs provided by actors and organizations, e.g., the green/blue areas made accessible for the care activity, coordinated activities between them, and the resulting outputs.

Analyzing the care service delivery chain where the GCIs have been integrated entails exploring whether and how health actors collaborate with others (e.g., owners of green/blue areas) to achieve expected outcomes. The provision of care services resulting from collaboration between health and non-health actors is facilitated through governance arrangements. For instance, formal agreements may be established where one party (e.g., healthcare institution) compensates for another (e.g., GC providers, green/blue area managers) for a set of care services directed at either the general population or specific user groups [69]. These services would include access to secure and suitable green or blue areas. Furthermore, analyzing the care service delivery chain in which the GCIs are integrated requires considering the environment and natural settings as key assets. These provide essential resources for effective GCIs, offering aesthetically pleasing green spaces, biodiversity, clean air, and soothing sounds, while also requiring proper management and protection. The service delivery chain leads to both health and environmental outcomes and impacts—referred to as the “Effects”—which generate feedback influencing both the “General settings” of the health governance system and the “Care Service Delivery” process.

While focusing on the care service delivery process can elucidate the upstream governance system, analyzing the multiple dimensions of the governance system can enhance the delivery of downstream care services and, potentially, the consequent health and environmental outcomes [60,61]. However, the access to and presence of people in natural settings as part of GCIs may negatively impact fauna, flora, and ecosystems. These impacts must be avoided or minimized when designing nature-based health interventions.

## 2. Material and Methods

**Figure 2:**
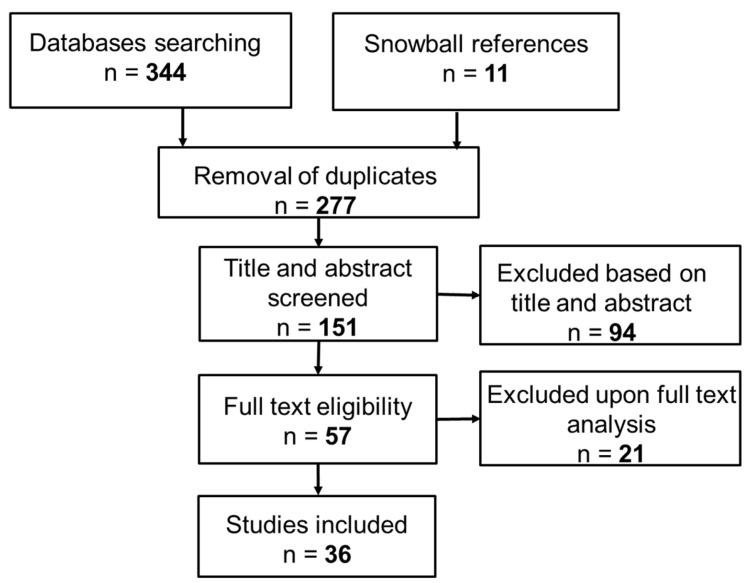
Prisma flow diagram illustrating the sequence of steps for the literature review.

Given the emerging nature of GC as a research field, we conducted a systematized literature review to synthesize available evidence on the integration of GCIs into conventional healthcare systems [61,69]. This review is essential to anchor potential future pathways for integration in the current knowledge of existing governance mechanisms, practices, and tools. It also helps identify which dimensions of health governance are already deemed relevant and applicable to GCIs, as well as those that have not yet been considered. The search process, inspired by Preferred Reporting Items for Systematic Reviews and Meta-Analyses (PRISMA) guidelines [70], is illustrated in Figure 2 and comprises three main phases: (i) search strategy for identifying relevant documents (including scientific articles, book chapters, conference papers, and scientific reports) through databases and other sources, (ii) screening process based on relevance and eligibility determined according to selection criteria, and (iii) final inclusion of documents to be analyzed.

### 2.1. Step (i): Search Strategy

To ensure comprehensive coverage of the transdisciplinary nature of our research topic, we employed a dual-database approach for document identification [71]. We utilized Clarivate Analytics’ Web of Science (WoS) and Elsevier’s Scopus search engines, leveraging their complementary strengths in covering natural and social sciences, respectively [72]. Our search strategy was refined through iterative attempts to identify the optimal combination of keywords. Ultimately, we developed three search strings representing the main research areas, which were then combined to ensure broad coverage of potentially relevant documents (Table 1).

The first search string addressed the concept of GC, acknowledging its status as an umbrella term encompassing diverse practices with varying interpretations across countries [20,73]. To account for this heterogeneity, we included a range of related terms, such as “therapeutic horticulture” or “care farming” (prevalent in the United Kingdom), “farm animal-assisted intervention” (common in Norway), and “social agriculture” (widespread in Italy and Spain) [74]. The second string focused on the healthcare sector, broadly defined to encompass the entire system of care services, including institutions, personnel, and processes involved in disease prevention, health promotion, and treatments. The third string explored the concept of governance, adopting an analytical perspective that considers both formal (institutional) and informal (behavioral) rules shaping actors’ interactions, with particular emphasis on governance arrangements [55,75,76]. We conducted searches in both Scopus and WoS databases during 2023 and the initial months of 2024, targeting article titles, abstracts, and keywords (topics for the WoS database). The database search was supplemented with additional documents identified through snowball referencing.

### 2.2. Step (ii): Screening and Eligibility

This consisted of two sub-steps: firstly, duplicated and/or incomplete references were removed; secondly, the documents were pre-screened by reading the title and abstract, excluding those unrelated. Subsequently, the pre-screened documents underwent further examination through a content-based relevance assessment. Eligibility for inclusion was attributed based on the following criteria: (i) mention of at least one GCI and (ii) presentation of direct or indirect references to governance concepts (e.g., organizational structure, types of arrangements, partnerships, collaborative efforts) or engagement of the healthcare providers and actors from other sectors. Conversely, publications were excluded from further analysis when (i) mentioning GC without linking to healthcare aspects, (ii) merely presenting clinical trial protocol results, or (iii) focusing exclusively on environmental aspects (e.g., architectural design or gardening techniques). No temporal restrictions were imposed for inclusion; however, articles were limited to those published in the English language. The research began on 24 January 2023, and the final search date across all databases was 5 February 2024. The first author conducted the pre-screening process and determined the eligibility of studies, while co-authors engaged in periodic reviews as part of an iterative approach to mitigate potential bias and ensure consistency in the selection process.

### 2.3. Step (iii): Final Inclusion of Documents

After thoroughly reading the content of the selected documents, we extracted relevant information from each study into an Excel database. The database comprised blocks related to bibliographic (e.g., title, author(s), year of publication, publication type, subject area) and general characteristics considered to be of interest, namely practices associated with GCIs, study design, and the first author’s country of origin. The methodology for qualitative analysis involved the extrapolation of evidence related to governance (see Appendix B), followed by a thematic synthesis approach. For the categorization process of governance dimensions, we drew inspiration from the schemes proposed by Aarons (2020) and Hox (1997) [77,78] for operationalizing theoretical concepts. We started with identifying main concepts or ideas (i.e., pieces of evidence related to governance), and then, we categorized these concepts according to the relevant dimensions and components of governance, aiming to highlight key aspects of the integration process.

## 3. Results

### 3.1. Descriptive Characteristics of the Selected Documents

**Figure 3:**
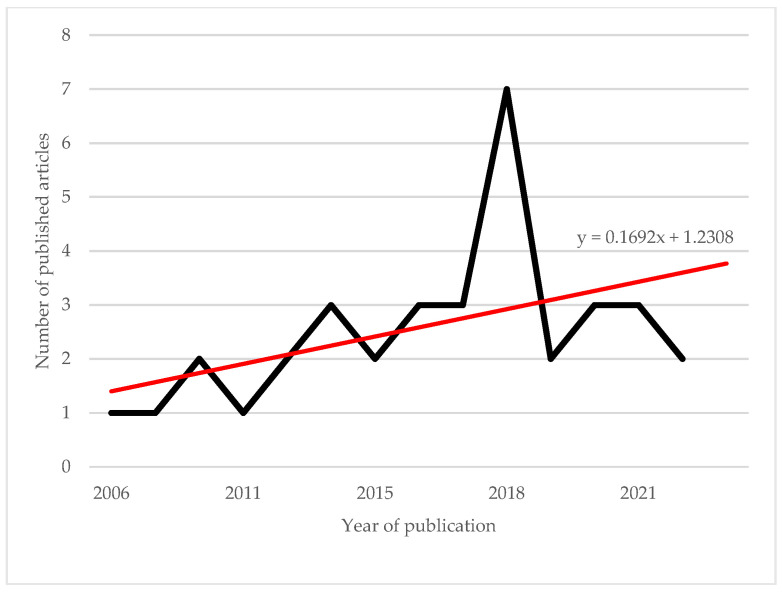
The number of published articles per year from 2006 to 2023 (Source: own elaboration based on Scopus and WoS databases).

**Figure 4:**
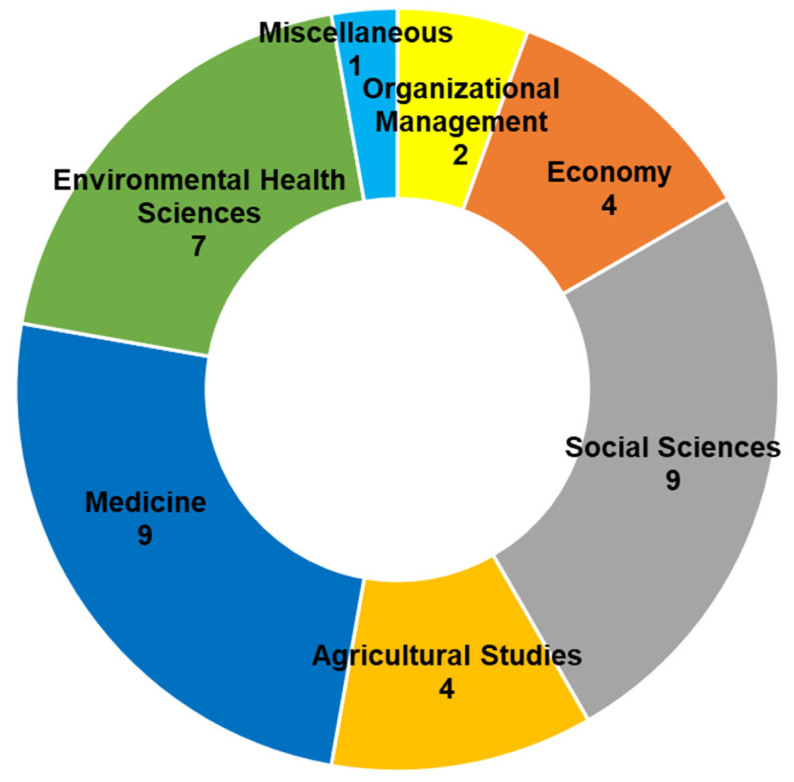
Subject areas of selected documents (Source: own elaboration based on Scopus and WoS databases).

Our search initially identified 344 documents, supplemented by 11 additional documents identified through snowball referencing (Figure 2). Following the screening process, 57 papers were assessed for eligibility, resulting in a final set of 36 documents spanning the period 2006–2024 (listed in Appendix A). The selected documents were classified by publication type: thirty-one scientific articles, two book chapters, two conference papers, and one scientific report. Regarding study design, qualitative research methods predominated (23 studies), employing techniques such as case study analysis and semi-structured interviews. Mixed methods were utilized in 11 studies, while only 2 studies employed purely quantitative methods, such as surveys and descriptive statistics. Our analysis reveals that research on this topic, published in English, is relatively recent, with the earliest publication dating to 2006 (Figure 3). As illustrated in Figure 4 and detailed in Appendix A, the selected articles span various journals and subject areas, primarily in the fields of Medicine (9), Social Sciences (9), and Environmental Health Sciences (7).

Analyzing the affiliation of the first authors reveals that, in recent years, the interest in integrating GCIs into the public health sector has spread internationally, particularly in some countries. Dutch (8) and Italian (7) authors have the highest count, followed by authors from the United States of America (4), the United Kingdom (4), and Spain, Norway, and Belgium, with three authors for each.

The analysis of the selected studies revealed various practices associated with GCIs, with “Social farming” and “Care farming” emerging as the dominant terms (Table 2).

### 3.2. An Ad Hoc Analytical Tool Based on Dimensions and Components of Governance

In an attempt to develop an ad hoc analytical tool to facilitate a common understanding and the sharing of best governance-related practices to guide actions towards integrating GCIs into conventional health systems, we identified concepts of governance that emerged from the literature review (see Appendix B) and categorized them by dimensions and components (Table 3). We ultimately identified four key dimensions of governance: (1) Organizational structure, (2) Knowledge, (3) Legitimacy, and (4) Decentralization. In categorizing governance-related concepts, we emphasized aspects relevant to GC actors, integrating them with those typically addressed in the governance of the health sector (see Figure 1). We recognize that our interpretations of the causal links between GCIs and governance may be influenced by personal assumptions. To mitigate this potential bias, efforts have been made through revision and comparison among the co-authors of the paper.

#### 3.2.1. Dimension 1—Organizational Structure

Across the continuum of care—from prevention to intervention—public health authorities are responsible for delivering essential services, including primary and specialist care. Generally, the delivery of health services can be conceptualized as an output intrinsically linked to the functioning of the healthcare system and its organizational structure.

Regardless of the specific services, the core of an organizational structure capable of providing them is comprised of the actors involved.

Integrating GCIs into the organizational structure of healthcare systems would introduce non-health actors and novel types of interactions into the service delivery chain. Consequently, this integration would generate a new combination of health services, governance processes, and arrangements.

Therefore, the first aspect to consider is the identification of key actors, their roles, and the nature of their interrelationships [61,85]. This understanding is crucial for effectively incorporating GCIs into existing healthcare structures and optimizing their potential benefits.

##### Component 1.1.—GC Actors and Their Roles

The roles of the GC actors can vary based on the type of provider, intervention, context specificities, and beneficiaries served. However, recurring roles within the network of involved actors can be observed. Our analysis identifies the following types of actors and their respective roles:

- Public authorities at various jurisdictional levels influence the decision-making processes and legitimize GCIs in institutional debates; they include political and government decision-makers.
- Health insurance companies relate to customers and offer health service packages aligned with client needs. Their roles may vary based on healthcare system characteristics.
- Private and voluntary sectors actors provide GCI-related services/products (e.g., farmers, landowners, entrepreneurs, NGOs).
- Care professionals in healthcare institutions are engaged in the design and implementation of GCIs and assist vulnerable subjects or groups during the activities; they serve as health service providers.
- GCI beneficiaries (clients or service users) are involved either through the healthcare facility they are affiliated with or by directly requesting the service.
- Researchers from various disciplines, including the medical field, psychology, and social science, contribute to GC knowledge advancement through scientific evidence.
- Third-sector organizations are generally seen as important contributors in the context of GCIs by supporting the initiative in different ways.
- Local communities constitute the sociocultural background with which many activities and actors involved in GCIs are intertwined.

Effectively addressing population health needs requires a complex blend of medical, scientific, technical, and political skills, and organizational requirements [56]. Thus, actors with diverse expertise can be viewed as governance inputs, with the linkages forming the operational core of governance processes. Accountability relationships in service delivery can be described as two-way relationships between actors and their linkages [58]. Stemming from these, the integration of GCIs into the healthcare system would imply reconfiguring the care service delivery chain by adding GC providers to health service providers and reshaping linkages with other actors, particularly governmental actors and GCIs beneficiaries. This implies establishing accountability of GC providers to politicians, users, and civil society [61,86]. The role of urban and land planners, nature conservation managers, and park authorities appears to be undervalued, as they are not prominently mentioned as key actors in the papers we reviewed. However, these stakeholders play a critical role in ensuring the provision of safe and accessible green and blue areas. Additionally, the interaction between these actors and the health sector seems to be an underexplored area in the existing literature, highlighting the need for further research on this topic.

##### Component 1.2.—Governance Approaches

**Figure 5:**
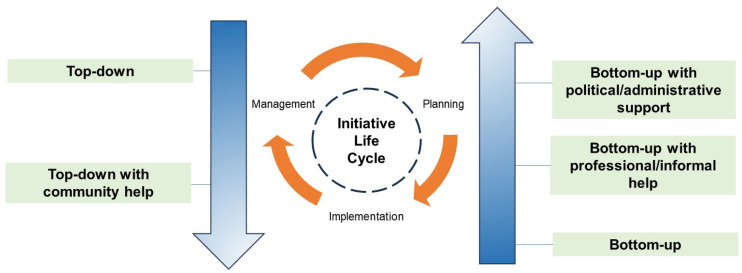
Governance approaches and community garden initiatives’ stages of development identified by [87] (Source: own elaboration).

Governance approaches characterize the structure of decision-making processes within the organizational unit under study. As noted in other fields, a continuum encompassing both top-down and bottom-up governance approaches can be observed also in GCIs. Fox-Kämper et al., (2018) [87] delved into this aspect by adapting McGlone’s (1999) [88] governance approaches to describe the governance structure for urban community gardens. When considering the different stages of development throughout the initiative life cycle, they noted that the implementation of community gardens typically begins with a top-down approach and then tends towards a community-based bottom-up approach during the management phase (Figure 5). Furthermore, the most common form of governance for the planning phase was represented by bottom-up approaches with political and/or administrative support. This support manifested in various forms such as land use permissions, financial resources, or technical advisory for initiative design. Notably, this support can extend into the implementation and management phases through continued donations or funding [87].

##### Component 1.3.—Models of Governance

The delivery of care services involving different actors necessitates ‘new’ models of governance, typically multi-level in nature. These models are characterized by both horizontal interactions, i.e., between public institutions and other actors, and vertical interactions, i.e., between different levels within the same organizational unit. Contrary to centralized decision-making processes, a multi-level governance model requires mediation between different interests and coordination between various levels and sectors of actors [89].

**Figure 6:**
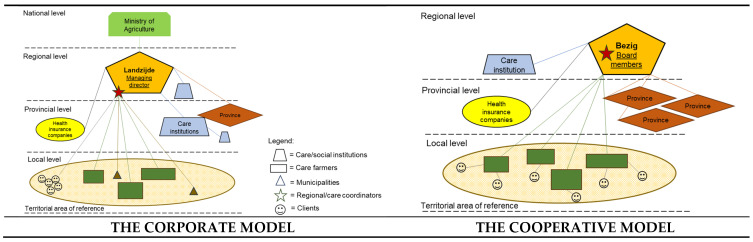
Governance organizational models related to the development of care farming in The Netherlands (Source: own elaboration based on [92]).

Adopting a multi-level analytical perspective—which recognizes the presence of multiple levels within the governance system of GCIs—allows for a broader understanding of actor interactions. These interactions can be represented as linkages occurring within or across scales (e.g., jurisdictional and institutional scales) [90]. These might be different depending on how actors interact across levels. Drawing inspiration from Hassink et al. (2016) [91], we have attempted to visually represent the corporate and cooperative models described for the development of care farming in The Netherlands. Figure 6 provides a graphical representation of the jurisdictional levels and types of actors involved.

#### 3.2.2. Dimension 2—Knowledge

Knowledge, a broad concept encompassing the acquisition of awareness, understanding, or information through experience or learning, plays a crucial role in governance processes. It influences actors’ actions and choices by shaping their attitudes, intentions, and decision-making processes (e.g., as described in the Theory of Planned Behavior [93]). Knowledge contributes to governance processes both directly, serving as a resource, instrument, or input, and indirectly by shaping actors’ preferences, methods of goal achievement, and network formation [94]. In our framework, knowledge is conceptualized as a determinant influencing institutional and individual behavior, consequently affecting both governance and organizational solutions related to GCIs.

##### Component 2.1.—Cultivate Awareness

For knowledge to effectively support governance processes, information should reach actors with both the interest and ability to use it effectively [60]. Awareness is considered a preliminary condition for knowledge acquisition. Our research has identified several challenges in this area.

First, a lack of awareness among healthcare professionals and patients was found regarding community gardens inside the clinics and their potential uses [95]. In general, healthcare professionals may be unaware of certain evidence if it is not presented in medical publications or other media typically consulted [96]. Second, healthcare professionals may be hesitant due to a lack of protocols on how to incorporate GCIs into medical advice or prescriptions, emphasizing the need to find pathways to translate research into practice [97]. In particular, information about green and social prescriptions is lacking due to the difficulties in reaching primary care professionals, such as general practitioners [98]. These aspects hinder the referral process, patient engagement, and accessibility to GCIs [99].

##### Component 2.2.—Knowledge Integration

Knowledge is normally dispersed among various actors within a governance system [100]. Our analysis of GCIs reveals a growing knowledge base concerning the health benefits derived from nature, which remains largely unrecognized by health actors and is inadequately integrated into medical practices. This divergence in perspectives can lead to conflicts and fragmentation within governance processes. To address these challenges, GC actors necessitate effective strategies for integrating diverse knowledge perspectives and for the success of the collaborative governance processes. Moreover, mobilizing the skills and competencies necessary for collaborative governance processes is required [101].

To promote knowledge integration across actors and disciplines at the organizational level, knowledge management strategies include developing training plans within healthcare institutions and organizing ’boundary experiences’ for actors with different frames [101].

The effective mobilization of skills and competencies can be achieved through several learning processes: (i) mediating between different competencies to integrate them into a common approach [102], (ii) facilitating communication between stakeholders by establishing a common vocabulary [98], (iii) implementing integrated training programs to share knowledge and competencies, such as joint training sessions for individuals with diverse backgrounds [102], and (iv) conducting demonstrative on-site activities and disseminating their results through various media channels [95].

##### Component 2.3.—Discourses

While the resources, role of the actors, and governance processes discussed thus far primarily concern the conditions for governance arrangements, policy ’discourses’—the views and narratives of the actors involved—relate to their ’substance’ [103,104].

Discourses play a significant role at two distinct levels. Firstly, they shape the interactions between the state, market, and civil society, analogous to relationships among policymakers, health providers, and clients/citizens [58]. Secondly, discourses influence actors’ perspectives on specific policy issues, including their views on problem interpretation, underlying causes, potential consequences, and available solutions [103]. Discourses can be translated into policies and programs at various levels of the institutional scale and intertwined across different sectors. The latest IUFRO report (2023) [105] on forest-based health initiatives notes that the intersection of forestry with other sectors generates a wide range of discourses permeating governance processes. Despite highlighting the essential contribution of forests to human health, the integration of these practices into the health governance system remains indirect or implicit.

Contrastingly, policy discourses around GCIs appear more mature within care farming. In this regard, Bock and Oosting (2010) [106] distinguished three discourses that inspire governance arrangements at the European level: (i) the discourse of multifunctional agriculture, (ii) the discourse of public health, and (iii) the discourse of social inclusion. These discourses characterize how care farming initiatives are interpreted, discussed, and organized, and which actors are involved throughout Europe. For example, in The Netherlands, care farming practices predominantly align with the discourse of multifunctional agriculture as part of farm income sources, while in Norway, Germany, and Austria, care farming is considered more within the public health discourse [107,108]. Differently, social inclusion is the central discourse in Italy [11,109], with care farming being seen as a facilitator of social reintegration and justice. Here, cooperatives often organize initiatives as part of their voluntary civic and political engagement. Also, in France and Ireland, civic and voluntary engagement drives GCI provision, organized by individual farmers and civic associations, generally without institutional support and formal regulations [11].

#### 3.2.3. Dimension 3—Legitimacy

The formal recognition of alternative and innovative interventions by public institutions and society necessitates legitimization [110]. Legitimacy assumes particular importance for the acceptance of GCIs by public and health authorities and civil society as complementary care opportunities to address the increasing demand for healthcare services.

Based on our analysis of the literature on GCIs, we have identified two key components of legitimacy: (i) “institutional legitimacy”, which refers to all conditions facilitating alignment with the requirements of the healthcare domain, and (ii) “innovative legitimacy”, where newcomers change the existing order and introduce novel elements to the sector in which they aim to operate [92,110,111].

##### Component 3.1.—Institutional Legitimacy

Institutional legitimacy is achieved when newcomers comply with certain field-specific assumptions about how a participant is expected to look and behave [111]. This concept is intrinsically linked to the recognition of an actor or organization within its operating field. For GC providers, considered newcomers in the health sector, this can correspond to their level of embeddedness in the organizational structure of the care domain. This level is characterized by the nature, depth, and extent of the actor’s ties to the network [112,113]. The highly institutionalized care sector typically restricts access to funding and imposes strict quality-related and administrative requirements on its organizations [114]. However, for a newcomer, being embedded in the network of the care sector offers several benefits, including exchange of knowledge, establishment of contacts, access to sources of evidence, availability of resources, information sharing, and support mechanisms [92]. Thus, for GC actors, the development of a professional identity consistent with institutional prescriptions can be beneficial.

For instance, it has been argued that the institutional legitimacy of social farming initiatives could be enhanced through the involvement of the academic world, the development of capacity-building processes, the establishment of quality standards, and the implementation of national legislative acts [115,116]. A concrete example of this process is evident in Belgium. In 2005, the Flemish government introduced legislation on care farming, allowing farmers to apply for a care farming subsidy from the Ministry of Agriculture to compensate for a loss of agricultural productivity. Simultaneously, to ensure institutional alignment, farmers are obligated to collaborate with healthcare facilities recognized by the Ministry of Public Health or with a counselling center for high school students under the Ministry of Education [14].

##### Component 3.2.—Innovative Legitimacy

Innovative legitimacy is gained when newcomers challenge the existing order in a field and introduce novel and valuable elements to the sector. De Clercq and Voronov (2009) [111] argue that this concept aligns with the change and innovation brought about by the creation of new businesses. In this regard, entrepreneurship is the actor’s innovative trait predominantly described within the study of the GCIs. Entrepreneurship in this context can be defined as the promotion of opportunity-driven behavior, crucial for generating and extracting value within and from the context [92]. Our literature review reveals that initiators of GCIs are often described as entrepreneurs, key actors who combine entrepreneurial spirit with adherence to institutional requirements. Regardless of their backgrounds, these entrepreneurs frequently possess trans-sectoral competencies and skills. These include the ability to collaborate across different sectors, the identification of customer needs, the formulation of effective strategies, ‘network orchestration’ (i.e., bringing diverse networks together), and fostering diversity and teamwork [117].

More broadly, it has been argued that innovative legitimacy can be acquired when GCIs gain people’s esteem, recognition, and credit. However, this can also occur when GCIs are perceived as cost-effective alternatives to conventional healthcare services [115,118]. Social farming, as a form of social entrepreneurship in the agricultural sector, exemplifies this principle of innovative legitimacy. It has changed public perceptions of farmers, farming practices, societal roles of agriculture, and the welfare state [9,119].

#### 3.2.4. Dimension 4—Decentralization

The trend towards decentralization in healthcare—characterized by the distribution of decision-making processes and health service delivery across local suppliers and stakeholders—has led to increased participation from various social actors. On the one hand, this shift challenges the central role of national health authorities, allocating more “decision space” to subnational and local authorities [58]; on the other hand, it recalls the need to harmonize health service provision [89]. Harmonization involves improving the complementarity of different providers and user groups to extend health coverage equitably and efficiency [89]. A mechanism to ensure such harmonization and complementarity is through the integration of the diverse entities operating within the system into health service delivery networks. These networks comprise professionals from various sectors collaborating to promote health [120]. Consequently, the current trend towards progressive decentralization in the health sector could facilitate the integration of GCIs into health service delivery networks. This integration has the potential to enhance individual and collective coping strategies by leveraging locally available resources (e.g., [121]) such as specific types of green or blue areas and natural settings that are unique to particular sites.

##### Component 4.1.—Public–Private Partnerships (PPPs)

Decentralization in healthcare affects governance processes at various levels and influences the organizational performance of actors along the care service delivery chain. The reshaping of relations towards a provision of care services tailored to local health needs offers the possibility of extending partnerships between the public and private sectors, as well as with other non-state organizations. In the context of GCIs, relationships between different actors may be formalized through the creation of Public–Private Partnerships (PPPs). Unlike traditional privatization or procurement, PPP arrangements divide responsibilities between public and private entities based on their respective strengths and capabilities. This approach allows for the participation of a broader range of stakeholders in the governance of PPP arrangements, including patients, NGOs, academic institutions, professional organizations, and religious organizations [122]. This broader participation ensures that PPPs within GC actors reflect the diverse needs and perspectives of the stakeholders involved. For instance, in Norway, farmers, municipal representatives, the Norwegian Labour and Welfare Organization, researchers, and care institutions collectively participated in initiatives to educate, motivate, and disseminate information about GCIs [123]. Private and public actors can effectively collaborate to provide locally led care services [124], as exemplified in the Friuli-Venezia Giulia Region in Italy, where the local health authority approached social cooperatives and private farmers to offer activities for their service users [125].

##### Component 4.2.—Boundary Spanners

The current trend in healthcare systems, shifting from hierarchical towards increasingly decentralized structures, is encouraging a transition to governance models aimed at creating and managing networks of actors [126]. As previously mentioned, one potential outcome of healthcare system decentralization is the creation of integrated health service delivery networks, i.e., a web of actors from different levels or sectors collaborating to promote health [120]. In the context of GCIs, these integrated networks comprise care professionals and actors working in the social, educational, and nature management sectors at different levels.

Due to the diversity of cultural values, beliefs, and stakes among GC actors, challenges in communication, collaboration, and mutual trust can emerge [127]. To address such difficulties, networks should include ‘boundary spanners’ [128]. In the context of GCIs, these actors play a crucial role in bridging organizations with potentially conflicting goals and expectations, acting as intermediaries [15,115,123]. Typically, boundary spanners in GCIs have a hybrid professional identity or background, adhering to multiple sets of values and practices [115]. For instance, in the development of care farming initiatives in The Netherlands, women have emerged as key boundary spanners. They demonstrate the ability to understand the needs and languages of multiple sectors and leverage their healthcare background to obtain support from care institutions [15]. Additionally, female farmers have introduced new elements to farms, including novel ways of thinking, cultural elements, logic, and rules, effectively integrating them into existing agricultural approaches [92,129].

## 4. Discussion and Recommendations

In this final section, based on our interpretation of the results, we propose practical recommendations for fostering the integration of GCIs into conventional healthcare systems. We outline the types of changes expected in current health governance structures to facilitate the adoption of nature-based health initiatives. These recommendations are derived from our comprehensive analysis of the literature and aim to provide actionable insights for policymakers, healthcare administrators, and GC practitioners.

### 4.1. Strengthening the Accountability of GC Actors in Healthcare Provision

For decades, international health organizations have advocated for the involvement of non-health actors at all levels within multi-sectoral and multi-actor approaches to healthcare provision (e.g., WHO, 1978, 1986, 2013). However, our analysis of GCIs, consistent with other public health studies [130,131], reveals limited evidence of this approach’s translation into everyday care practices. This suggests that GC actors are not yet adequately recognized as healthcare providers, and collaborative efforts to date have primarily stemmed from casual and informal relationships. Moreover, actors who are often overlooked but are crucial to GCIs, such as managers of green/blue areas, parks, and natural settings, landowners, and urban and landscape planners, i.e., those responsible for the quality and safety of the outdoor spaces where healthcare activities can be promoted, should be intercepted and actively involved in the design of care provision. To address this issue, we propose strengthening the accountability of GC actors to other societal stakeholders [86]. One potential approach involves reconfiguring care service delivery through contract-like arrangements that specify the roles and responsibilities of various parties [22,132]. To this end, the formalization of multilateral agreements should be integrated into national strategies for disease prevention and health promotion, and recognized within the institutional framework at each governance level. Concurrently, from the users’ perspective, accountability should be conceptualized as a mechanism for holding GC actors responsible for achieving objectives related to improved health outcomes, access to quality services, and patient satisfaction [86,133], while simultaneously safeguarding the environment from potential negative impacts.

### 4.2. Valuing the Role of Local Government Authorities and Intermediary Organizations in Creating New Integrated Delivery Networks

The integration of GCIs into prevention and health promotion strategies should involve actors at various levels of the institutional and territorial scales. This suggests the importance of vertical and horizontal interactions among actors [134,135] and the need for designated coordinators to manage trade-offs, mitigate potential conflicts, and steer synergies across different levels. Corroborated by studies in related fields [136,137], government authorities closest to the local territory, such as provinces and municipalities, could assume this coordination role in synergy with health authorities. Local authorities can, for instance, identify and ensure the adequate management of the green or blue areas for GCI implementation. This can happen by mediating with landowners through the constitution of Public–Private Partnerships (PPPs), e.g., for private green area rentals and by engaging in dialogue with researchers regarding criteria for identifying suitable areas. Furthermore, public authorities and health institutions should aim to establish integrated delivery networks to efficiently distribute GC services across the territory. This can be achieved by facilitating exchange among actors and resolving disagreements [138,139]. Our findings highlight the importance of recognizing and promoting the role of boundary spanners to facilitate networking and dialogue among practitioners and institutional actors. Universities, as knowledge mobilization centers, can serve as intermediary organizations by raising awareness, facilitating access, and transferring research results to practitioners and decision-makers in the field [140].

### 4.3. Integrating Different Disciplines and Knowledge Perspectives

Our analysis underscores the importance of bringing together different perspectives and knowledge across actors and disciplines to facilitate the integration of GCIs into conventional health systems. For instance, economic aspects are relevant: the potential of GCIs to reduce public health costs should be further considered among care professionals and policymakers [141]. In this regard, the adoption of “green” or “nature” prescriptions should be considered as a referral pathway to GCIs [142]. The establishment of protocols, guidelines, and a list of locally accredited GC providers would facilitate the integration of GCIs into conventional care pathways [143]. For promoting knowledge integration, researchers should gather empirical evidence on factors influencing the effectiveness of GCIs [144], and concentrate their efforts on identifying the most effective and economically viable programs and determining the best tools for measuring program effectiveness [145,146,147]. The monitoring of GC programs should also systematically integrate measures of the environmental impacts of the initiatives on green/blue areas and their natural elements, such as fauna, flora, and ecosystem stability, as these aspects are currently not considered. Addressing this gap would require the incorporation of ecology-related expertise. Additionally, the use of participatory approaches could help deepen the understanding of the effects on community well-being and resilience [148,149,150]. Educational actors should explore opportunities to incorporate GC-related topics into school programs or establish additional educational courses to train qualified GC professionals (e.g., [151,152]). GC providers should intensify efforts to communicate the benefits of GCIs for health, well-being, and social inclusion to the wider public while increasing general awareness about the activities. Insurance companies should propose targeted GCIs to clients and collaborate with GC providers and healthcare institutions to develop innovative services. Policymakers should enhance the visibility of GCIs by creating platforms for knowledge transfer. Furthermore, public local authorities should encourage citizens’ participation in GCIs, e.g., through direct engagement in the management of green and blue spaces. To enhance collaboration between health and environmental sectors, a mutual exchange of expertise is crucial. Health professionals should educate nature management experts and landowners about human health systems, including the potential risks and challenges individuals may face when interacting with nature. Conversely, environmental specialists should inform healthcare providers about the functioning and fragile equilibrium of green and blue spaces and ecosystems. This cross-disciplinary knowledge sharing would foster a deeper understanding of human health needs, the potential of natural settings, and environmental concerns. Ultimately, such an exchange could promote a more holistic approach to health and nature management, encouraging greater care for the environment while maximizing the health benefits of natural settings.

### 4.4. Evidencing the Effectiveness of Introducing GCIs Since the Beginning, Not Only on Outcomes in Terms of Human Well-Being

Our analysis supports the potential of GCIs as a means for healthcare institutions to outsource services effectively. The resulting care service delivery model, at the level of individual actors or organizations, necessitates a reconfiguration of both the organizational structure and the physical location of activities, while maintaining a clear focus on achieving the expected health outcomes (performance objectives) [153].

Adopting a Theory of Change (ToC) perspective underscores the importance of evaluating performance at the initiative level across various developmental stages (e.g., planning, implementation, and management phases), as suggested by [87]. This approach can demonstrate the relevance, effectiveness, and efficiency of GCIs (e.g., [154]), providing important information for policymakers and practitioners regarding decisions and actions to integrate these initiatives into conventional healthcare settings. While GCIs primarily aim to promote human well-being, their design and implementation should simultaneously prioritize the preservation of environmental health, the protection of ecosystems, and the fostering of awareness regarding the intrinsic value of nature and its benefits [155]. Consequently, evaluating the changes achieved through GCIs should employ a comprehensive approach that considers their societal and social embeddings, as observed in other research fields. For example, participatory evaluation methods have been effectively applied to the monitoring of Nature-Based Solutions (NbSs) [156]. Such participatory approaches draw on a range of techniques from the social sciences and humanities, including in-depth interviews, focus groups, observations, and workshops [157]. These techniques allow for the capturing of data on co-benefits across various sectors, as well as the context-level changes such as perceptions, experiences, and practices. This bottom-up methodology provides an opportunity to develop community-driven and sustainability indicators at a local scale. It clarifies the relationship between NbSs and their immediate environment, thereby informing the decision-making process [157,158].

Similarly, for GCIs, impact analysis is essential for developing improvement strategies and enabling a comprehensive longitudinal understanding of their effects [159]. This understanding necessitates co-creating a ToC to guide indicator selection and recommends a transdisciplinary approach and social engagement.

### 4.5. Strengthening the Inclusiveness of Non-Health Actors and the Credibility of GC Providers

Legitimacy is a crucial dimension within the governance of GCIs, particularly concerning the inclusiveness of non-health actors and the credibility of GC providers in delivering health services within institutional and social contexts [55,58]. Therefore, policymakers should strive for institutional recognition and legal clarity regarding the roles and responsibilities of GC actors at all levels, thus creating conditions for non-health actors to be accepted as alternative providers of care services. At the same time, to be legitimized by government institutions, GC providers should demonstrate credibility as professionals by effectively responding to population health needs and provide evidence of tangible results to gain legitimacy from government institutions [58].

The evolution of social farming initiatives in certain countries exemplifies how inclusiveness, tied to institutional legitimacy, can be promoted through legislative acts, accreditation systems, and national funding support [15,92,160]. Therefore, governments should assist GC initiators in adhering to institutional requirements and mitigate potential economic losses by allocating resources to GC providers.

## 5. Conclusions

While extensive research exists on the multiple benefits of GCIs for human health and well-being, evidence on governance aspects—such as actors’ roles, relationship types, and factors influencing collaboration processes—is limited.

In response to this scarcity of governance-related research, we conducted a systematized literature review to identify these aspects and possible changes that are necessary for integrating GCIs into conventional health systems.

In response to the first guiding question, “What are the key governance dimensions?”, we identified four governance dimensions: organizational structure, knowledge, legitimacy, and decentralization. These dimensions comprise various aspects, which we grouped into ten components. Given the intersectoral nature of GCIs, it is likely that additional dimensions are needed, particularly those related to the economic and ecological implications of GCIs. Regarding the second guiding question, “Who are the relevant actors involved?”, we found that healthcare professionals hold the most prominent roles. Other actors—such as landowners, urban and landscape planners, and green and blue area managers—play a minor role and have limited visibility. These actors should be explicitly involved from the outset to ensure that GCIs are effective, safe, and well-integrated into conventional healthcare systems. Finally, in addressing the third research question, “What governance changes are required for the mainstreaming of GCIs into conventional health systems?”, we identified the following needs:

1. Strengthening the accountability of GC actors in healthcare provision and explicitly including green and blue area/resource managers in the process.
2. Valuing the role of local government authorities and intermediary organizations in creating new integrated service delivery networks.
3. Integrating diverse disciplines and knowledge perspectives to foster a deeper understanding of human health needs, the potential of natural settings, and environmental concerns.
4. Demonstrating the effectiveness of GCIs from their inception, focusing not only on outcomes related to human well-being, but also on environmental impact.
5. Enhancing the inclusiveness of non-health actors and improving the credibility of GC providers.

### Limitations and Directions for Future Research

We proposed a conceptual framework for integrating GCIs into health governance and designed an analytical tool based on the relevant dimensions and components of governance. We believe this combination could pave the way for developing a set of indicators that would serve as valuable tools for guiding stakeholders and decision-makers in designing and evaluating key governance aspects, which are an essential part of the process of mainstreaming GCIs. While some countries, including Japan, South Korea, the USA, Canada, and New Zealand, have already made progress in this direction [16,17,18,161,162], key governance aspects have yet to be systematically identified and organized to guide other nations towards similar advancements. In this sense, we believe our framework represents “a first step to give governance analysis greater explanatory power and to therefore increase its potential for having empirical applicability” [56] (p. 8). However, it is important to note that the set of governance dimensions identified in this work, i.e., organizational structure, knowledge, legitimacy, and decentralization, should be regarded as neither exhaustive nor fixed. On the contrary, we hope it can serve as a starting point for future research and policy actions. This limitation is partly attributable to the selection of documents being restricted to English-language studies and the exclusion of grey literature, which may have constrained the diversity of the governance perspectives and examples included. Moreover, GC, as an innovative and multisectoral frontier of research and healthcare, likely requires additional governance dimensions beyond the canonical ones typically applied in the health sector. For example, further insights are needed into the ecological aspects, with indicators and dimensions directly related to (i) impacts on ecosystems; (ii) economic aspects, with indicators capable of estimating the cost versus benefits of GCIs; and (iii) specific legal aspects, such as regulations surrounding property rights, assurances, and responsibilities, to prevent conflicts in the event individuals are injured after accessing a green or blue area. We propose that future research could combine typical healthcare governance dimensions with those applied in other sectors, such as natural resource management or network rural governance. Moreover, the role of urban and land planners, nature conservation managers, and park authorities, as well as the interactions between these actors and those from the health sector, should be further explored in future research. For instance, aspects like the actors’ network structure and density could be explored using Social Network Analysis techniques. We also acknowledge that this study provides an initial overview of potential actions needed to foster the integration of GCIs into conventional healthcare settings, thus bringing more nature into everyone’s lives. A crucial next step in gaining insights into its practical applicability would be to empirically validate this work in real-life settings. This could be achieved by examining whether and how these actions have been implemented, and to what extent, in countries where GCIs have already been integrated into conventional health systems.

## Figures and Tables

**Table 1:** Search strings used to query bibliographic databases.

Thematic Area	Boolean Term	Keywords
Green Care		“green care” OR “nature care” OR “nature therap*” OR “green therap*” OR “wilderness therap*” OR “nature-based care” OR “nature-based therapy” OR “ecotherapy” OR “forest care” OR “forest-based therapy” OR “woodland therapy” OR “green exercise” OR “green gym” OR “green recreation” OR “restorative activit*” OR “outdoor therap*” OR “healing landscape*” OR “rehab* garden” OR “nature-based rehabilitation” OR “nature based rehabilitation” OR “therap* garden*” OR “healing garden*” OR “therapeutic horticulture” OR “social horticulture” OR “gardening” OR “horticultur* therap*” OR “care farm*” OR “social farm*” OR “animal-assisted therap*” OR “animal-assisted activit*”
Healthcare	AND	“healthcare” OR “health-care” OR “health care” OR “health system”
Governance	AND	“govern*” OR “governance framework” OR “governance dimension*” OR “multi-level” OR “trans-sectoral*” OR “cross-sectoral*” OR “network*” OR “alliance*” OR “arrangement*” OR “collaboration*” OR “instit*” OR “collab*” OR “agreement*” OR “synerg*”

**Table 2:** Practices associated with GCIs that emerged from the analysis (Source: own elaboration based on Scopus and WoS databases).

Practices Associated with GCIs	Number of Studies	Suggested Definition as Reported in the Paper (Citation)
Social farming	12	“…a social innovation process that mobilizes resources—from agricultural and rural areas—to respond to local social needs that the state and the market are unable to meet.” [79]; p. 100
Care farming	9	“the use of commercial farms and agricultural landscapes as a base for promoting mental and physical health, through normal farming activity.” [80]; p. 19
Community gardening	5	“open spaces which are managed and operated by members of the local community in which food or flowers are cultivated.” [81]; p. 364
Therapeutic gardening	1	“a plant-dominated environment purposefully designed to facilitate interaction with the healing elements of nature.” [82]
Green Care	4	“an umbrella term for a broad spectrum of health-promoting interventions that all use both biotic and abiotic elements of nature in their treatments. The ultimate goal is to maintain or promote a person’s social, physical, mental, and even educational well-being.” [12]; p. 106
Ecotherapy	1	“the practice of supporting vulnerable people (e.g., those with disabilities or mental health needs), to work with nature (both plants and wildlife), with the specific aim of the conservation or establishment of a local habitat or green space as a form of therapy.” [10]; p. 29
Forest-based initiatives	1	“organized initiatives, encompassing everything from single stand-alone activities to national programs, which can be both for-profit and not-for-profit, and that use (passively or actively) contact with a forest’s elements and atmosphere to increase the level of wellbeing of individuals, people, and communities.” [83]; p. 3
Nature-based care	1	“an umbrella term for health care interventions related to nature, such as green prescriptions, nature-based health interventions, nature-assisted therapies, and green care.” [48]; p. 2
Animal-assisted interventions (AAIs)	2	“any intervention that intentionally includes or incorporates animals as part of a therapeutic or ameliorative process or milieu.” [84]; p. 36

**Table 3:** Governance dimensions and components (Source: own elaboration).

Governance Dimensions	Governance Components
1. Organizational structure	1.1. GC actors and their roles
	1.2. Governance approaches
	1.3. Models of governance
2. Knowledge	2.1. Cultivate awareness
	2.2. Knowledge integration
	2.3. Discourses
3. Legitimacy	3.1. Institutional legitimacy
	3.2. Innovative legitimacy
4. Decentralization	4.1. Public–private partnerships (PPPs)
	4.2. Boundary spanners

## Data Availability

No new data were created or analyzed in this study.

## Appendix A

**Table A1:** Publications included in the systematized review.

N°	Title	Authors	Publication Year	Source Title	Subject Area
1	Designing and Implementing Animal-Assisted Therapy Programs in Health and Mental Health Organizations	Mallon G.P., Ross S.B., Ross L. [133]	2010	Handbook on Animal-Assisted Therapy	Psychology
2	Green care governance: Between market, policy and intersecting social worlds	Vik J., Farstad M. [123]	2009	Journal of Health, Organisation and Management	Health organization and management research
3	Developing a novel health and well-being service: The value of utilising the restorative benefits of nature in the UK	Custance P., Hingley M., Wilcox D. [124]	2011	Journal of Marketing Management	Marketing research
4	Multifunctionality and care farming: Contested discourses and practices in Flanders	De Krom M.P.M.M., Dessein J. [14]	2013	NJAS—Wageningen Journal of Life Sciences	Agricultural and Life Sciences
5	Multifunctional Agriculture Meets Health Care: Applying the Multi-Level Transition Sciences Perspective to Care Farming in the Netherlands	Hassink J., Grin J., Hulsink W. [117]	2013	Sociologia Ruralis	Social Sciences
6	Farming with care: The evolution of care farming in the Netherlands	Hassink J., Hulsink W., Grin J. [15]	2014	NJAS—Wageningen Journal of Life Sciences	Agricultural and Life Sciences
7	Development policies for social farming in the EU-2020 strategy	Scuderi A., Timpanaro G., Cacciola S. [163]	2014	Quality—Access to Success	Business, Management and Accounting
8	Outsourcing Mental Health Care Services? The Practice and Potential of Community-Based Farms in Psychiatric Rehabilitation	Iancu S.C., Zweekhorst M.B.M., Veltman D.J., van Balkom A.J.L.M., Bunders J.F.G. [164]	2015	Community Mental Health Journal	Public sector mental health services (Community Psychiatry)
9	Green Care’ in Poland -Application of horticulture for improvement of human life quality and environment protection	Latkowska M.J. [102]	2015	Acta Horticulturae	Horticultural Science—Agricultural and Biological Sciences
10	Entrepreneurship in agriculture and healthcare: Different entry strategies of care farmers	Hassink J., Hulsink W., Grin J. [91]	2016	Journal of Rural Studies	Social Sciences
11	Identity formation and strategy development in overlapping institutional fields: Different entry and alignment strategies of regional organizations of care farms into the healthcare domain	Hassink J., Grin J., Hulsink W. [92]	2016	Journal of Organizational Change Management	Organizational Change Management and development
12	Social farming: a proposal to explore the effects of structural and relational variables on social farm results	Bassi I., Nassivera F., Piani L. [165]	2016	Agricultural and Food Economics	Agricultural Economics
13	Enriching the multi-level perspective by better understanding agency and challenges associated with interactions across system boundaries. The case of care farming in the Netherlands: Multifunctional agriculture meets health care	Hassink J., Grin J., Hulsink W. [129]	2018	Journal of Rural Studies	Social Sciences
14	Farm-based day care in Norway—A complementary service for people with dementia	Ibsen T.L., Eriksen S., Patil G.G. [166]	2018	Journal of Multidisciplinary Healthcare	Multidisciplinary Healthcare—Nursing
15	It’s not therapy, it’s gardening’: Community gardens as sites of comprehensive primary healthcare	Marsh P., Brennan S., Vandenberg M. [167]	2018	Australian Journal of Primary Health	Medicine–community health services and primary healthcare
16	Nature-based interventions for mental health care: Social network analysis as a tool to map social farms and their response to social inclusion and community engagement	Borgi M., Marcolin M., Tomasin P., Correale C., Venerosi A., Grizzo A., Orlich R., Cirulli F. [125]	2019	International Journal of Environmental Research and Public Health	Environmental Research and Public Health
17	Characteristics and challenges for the development of nature-based adult day services in urban areas for people with dementia and their family caregivers	Hassink J., Vaandrager L., Buist Y., de Bruin S. [114]	2019	International Journal of Environmental Research and Public Health	Environmental Research and Public Health
18	Social farming as an innovative approach to promote mental health, social inclusion and community engagement	Borgi M., Collacchi B., Correale C., Marcolin M., Tomasin P., Grizzo A., Orlich R., Cirulli F. [97]	2020	Annali dell’Istituto Superiore di Sanita	Medicine
19	Let nature be thy medicine: A socioecological exploration of green prescribing in the UK	Robinson J.M., Jorgensen A., Cameron R., Brindley P. [98]	2020	International Journal of Environmental Research and Public Health	Environmental Research and Public Health
20	Farm-based day care on the market: The case of dementia care services in Norway	Farstad M., Logstein B., Haugen M.S., O’Connor D. [168]	2021	Journal of Rural Studies	Social Sciences
21	Therapeutic Community Gardening as a Green Social Prescription for Mental Ill-Health: Impact, Barriers, and Facilitators from the Perspective of Multiple Stakeholders	Wood C.J., Polley M., Barton J.L., Wicks C.L. [99]	2022	International Journal of Environmental Research and Public Health	Environmental Research and Public Health
22	Healthy Living Cambridge Kids: A Community-based Participatory Effort to Promote Healthy Weight and Fitness	Chomitz, VR; McGowan, RJ; Wendel, JM; Williams, SA; Cabral, HJ; King, SE; Olcott, DB; Cappello, M; Breen, S; Hacker, KA [150]	2010	Obesity	Medicine
23	Process Evaluation of a Community Garden at an Urban Outpatient Clinic	Milliron, BJ; Vitolins, MZ; Gamble, E; Jones, R; Chenault, MC; Tooze, JA [95]	2017	Journal Of Community Health	Community Health
24	Social farming in Catalonia: Rural local development, employment opportunities and empowerment for people at risk of social exclusion	Guirado, C; Valldeperas, N; Tulla, AF; Sendra, L; Badia, A; Evard, C; Cebollada, A; Espluga, J; Pallares, I; Vera, A [74]	2017	Journal Of Rural Studies	Social sciences
25	Farming for Life Quality and Sustainability: A Literature Review of Green Care Research Trends in Europe	Garcia-Llorente, M; Rubio-Olivar, R; Gutierrez-Briceno, I [7]	2018	International Journal Of Environmental Research And Public Health	Environmental Research and Public Health
26	Social return and economic viability of social farming in Catalonia: a case-study analysis	Tulla, AF; Vera, A; Valldeperas, N; Guirado, C [141]	2018	European Countryside	Rural research
27	Nature’s Contributions to Human Health: A Missing Link to Primary Health Care? A Scoping Review of International Overview Reports and Scientific Evidence	Lauwers, L; Bastiaens, H; Remmen, R; Keune, H [48]	2020	Frontiers In Public Health	Medicine—Public Health
28	Extending the concept of social farming: Rural development and the fight against organized crime in disadvantaged areas of southern Italy	Elsen, S; Fazzi, L [79]	2021	Journal Of Rural Studies	Social sciences
29	The Cost Effectiveness of Ecotherapy as a Healthcare Intervention, Separating the Wood from the Trees	Hinde, S; Bojke, L; Coventry, P [144]	2021	International Journal Of Environmental Research And Public Health	Environmental Research and Public Health
30	Sustainability capacity of a vegetable gardening intervention for cancer survivors	Cases, MG; Blair, CK; Hendricks, PS; Smith, K; Snyder, S; Demark-Wahnefried, W [159]	2022	Bmc Public Health	Medicine—Public Health
31	The Economics of Green Care in Agriculture	Dessein, J. and B.B. Bock, eds. [73]	2010	COST Action 866, Green Care in Agriculture	Economy
32	Transition Management and Social Innovation in Rural Areas: Lessons from Social Farming	Di Iacovo, F. Moruzzo, R. Rossignoli, C. Scarpellini, P. [118]	2014	Journal of Agricultural Education and Extension	Social sciences
33	The development of social farming in Italy: A qualitative inquiry across four regions	M. Dell‘Olio, J. Hassink and L. Vaandrager [115]	2017	Journal of Rural Studies	Social sciences
34	Urban community gardens: An evaluation of governance approaches and related enablers and barriers at different development stages	Runrid Fox-Kämper, Andreas Wesener, Daniel Münderlein, Martin Sondermann, Wendy McWilliam, Nick Kirk [87]	2018	Landscape and Urban Planning	Social sciences
35	The Italian Agreement between the Government and the Regional Authorities: National Guidelines for AAI and Institutional Context	Simonato, M.; De Santis, M.; Contalbrigo, L.; Benedetti, D.; Finocchi Mahne, E. [116]	2018	People and Animals: The International Journal of Research and Practice (PAIJ)	Psychology
36	Forests and Trees for Human Health: Pathways, Impacts, Challenges and Response Options. A Global Assessment Report	Cecil Konijnendijk, Dikshya Devkota, Stephanie Mansourian & Christoph Wildburger (eds.) [105]	2023	International Union of Forest Research Organizations (IUFRO) World Series Volume 41.	Forestry

## Appendix B

**Table A2:** Summary of key characteristics of included studies.

N°	Study Design	First Author’s Country of Origin	Governance Aspects Mentioned	Database
1	Qualitative	USA	Administrative organizational rules	Scopus
2	Qualitative	Norway	Corporativism, public–private partnerships, network governance, multi-level, multi-arena concepts	Scopus
3	Qualitative	United Kingdom	Public–private partnerships, network approach	Scopus
4	Qualitative	Belgium	Governance arrangements, cross-sectoral dynamics	Scopus
5	Qualitative	The Netherlands	Arrangements with health insurance companies, organizational arrangements, institutional arrangements	Scopus
6	Qualitative	The Netherlands	Subcontracting arrangements, financial arrangements	Scopus
7	Qualitative	Italy	Multi-sector and multi-actor approach, institutional acknowledgment, local networks, bottom-up approach	Scopus
8	Quantitative and qualitative	The Netherlands	Service characteristics, service organization, links with local communities, various organizational forms	Scopus
9	Qualitative	Poland	Multi-sector approach	Scopus
10	Quantitative and qualitative	The Netherlands	Financial arrangements, authorization arrangements, institutional arrangements	Scopus
11	Qualitative	The Netherlands	Institutional arrangements, formal arrangements	Scopus
12	Quantitative and qualitative	Italy	Structural and relational variables, multi-sector approach, networks	Scopus
13	Qualitative	The Netherlands	Institutional arrangements	Scopus
14	Quantitative and qualitative	Norway	Collaborations among actors, accountability, multi-sector approach	Scopus
15	Qualitative	Australia	Collaborations among actors, multi-sector approach, synergies, multidisciplinary services	Scopus
16	Quantitative and qualitative	Italy	Public–private partnerships	Scopus
17	Quantitative and qualitative	The Netherlands	Collaborations among actors, challenges, and facilitators to the implementation of nature-based day services in care organization	Scopus
18	Quantitative and qualitative	Italy	Hybrid governance models	Scopus
19	Quantitative and qualitative	United Kingdom	Transdisciplinary collaborations	Scopus
20	Qualitative	Norway	Public subsidy arrangements	Scopus
21	Qualitative	United Kingdom	Barriers and facilitators to referral and uptake from the perspectives of multiple stakeholders	Scopus
22	Quantitative and qualitative	USA	Multicomponent intervention, social–ecological model	Web of Science
23	Quantitative	USA	Engagement of healthcare providers	Web of Science
24	Quantitative and qualitative	Spain	Engagement of environmental, health, educational, and social sectors	Web of Science
25	Qualitative	Spain	Multi-sector approach, credibility, social capital	Web of Science
26	Quantitative and qualitative	Spain	Multi-sector and multi-actor approaches	Web of Science
27	Qualitative	Belgium	Multi-actor approach, interdisciplinary collaborations, the role of the local authorities, bottom-up approaches, and horizontal networks	Web of Science
28	Qualitative	Italy	Governance models of the different organizations (associates, volunteers, staff members associates, volunteers, and members of other civil society organizations)	Web of Science
29	Qualitative	United Kingdom	Potential costs and benefits of ecotherapy, clinical and cost-effectiveness	Web of Science
30	Quantitative	USA	Individual and organizational (program) sustainability, organizational capacity	Web of Science
31	Qualitative	Belgium	Entrepreneurial, financial arrangements	Snowball referencing
32	Qualitative	Italy	Institutional arrangements, co-production, cooperation	Snowball referencing
33	Qualitative	The Netherlands	Multi-level and multi-sector approaches, networks, legitimacy	Snowball referencing
34	Qualitative	Germany	Cooperative forms of governance, top-down, top-down with community help, bottom-up with professional help, bottom-up with informal help, bottom-up, bottom-up with political and/or administrative support, legislative act	Snowball referencing
35	Qualitative	Italy	Institutional agreement, legislative act, agreement and guidelines (soft law), legislative regulation	Snowball referencing
36	Qualitative	Austria	Governance arrangement, network governance	Snowball referencing
